# 

**Published:** 1861-03

**Authors:** 


### CHICAGO MEDICAL JOURNAL.

**Terms, $2.00 per Annum.**

### CONTENTS OF THE MARCH NUMBER.

SELECTED.

| | Page. |
|---|---|
| Treatment of Blenorrhœa and Kindred Affections | 129 |
| Ergot; its Natural History and Uses as a Therapeutic Agent | 133 |
| On Glycosuria as an Accompaniment of Marsh Fevers. By Dr. Burdel, Physician to the Vierzon Hospital | 135 |
| Cutaneous Discolorations | 136 |
| Hypochondriacal Insanity as a Precursor of General Paralysis. By M. Baillarger, of the Salpetriere, Paris | 139 |
| Physiology and Scepticism. Brinton's Lecture—Extract | 144 |
| ORIGINAL COMMUNICATIONS. | |
| Valedictory to the Graduates of Rush Medical College, Feb. 27th, 1861. By J. Adams Allen, A. M., M. D., Prof. Prin. and Prac. of Med., and Clin. Med | 147 |
| Inversion of the Womb. Case Reported to the Chicago Academy of Medical Sciences, Feb. 1st, 1861. By R. C. Hamill, M. D | 167 |
| Protective Power of Vaccination. By H. Noble, M. D | 173 |
| Foreign Bodies on the Cornea. By E. L. Holmes, M. D., of Chicago | 177 |
| Miasmatic Hæmaturia. By J. Low, M. D., McGregor, Iowa | 181 |
| Proceedings of Societies | 184 |
| EDITORIAL. | |
| Editorial and Miscellaneous | 187 |

